# Keratin protein nanofibers from merino wool yarn: a top-down approach for the disintegration of hierarchical wool architecture to extract α-keratin protein nanofibers†

**DOI:** 10.1039/d3ra07063h

**Published:** 2024-02-23

**Authors:** Nadeeka D. Tissera, Ruchira N. Wijesena, Natali Ludowyke, Gayan Priyadarshana, Damayanthi Dahanayake, Rohini M. de Silva, K. M. Nalin de Silva

**Affiliations:** a Institute of Technology, University of Moratuwa Diyagma Homagama Sri Lanka nadeekat@itum.mrt.ac.lk +94 71 4044269; b Sri Lanka Institute of Nanotechnology Nanotechnology & Science Park, Mahenwatta, Pitipana Homagama Sri Lanka; c Faculty of Technology, University of Sri Jayewardenepura Pitipana Homagama Sri Lanka; d Faculty of Science, NSBM Green University Mahenwaththa, Pitipana Homagama Sri Lanka; e Centre for Advanced Materials and Devices (CAMD), Department of Chemistry, Faculty of Science, University of Colombo Colombo Sri Lanka

## Abstract

We report the extraction of keratin nanofibers from the medulla of a parent yarn after denaturing the cuticle and cortex microstructures of a merino wool yarn. Controlled alkaline hydrolysis, followed by high-speed blending in acetic acid, allowed for the extraction of keratin protein nanofibers with an average diameter of 25 nm and a length of less than 3 μm. SEM and AFM analyses showed the removal of cuticle cells from the yarn. FT-IR and DSC analyses confirmed the hydrolysis and denaturation of the sheet protein matrix of cuticle cells. XPS analysis provided strong evidence for the gradual removal of the epicuticle, cuticle cells, and cortex of the hierarchical wool structure with an increase in alkaline hydrolysis conditions. It was confirmed that the merino wool yarn subjected to hydrolysis under alkaline conditions exposed its internal fibrillar surface. In an acetic acid medium, these fibrillar surfaces obtained a surface charge, which further supported the defibrillation of the structure into its individual nanofibrils during high-speed blending. The extracted nanostructures constitute mainly α-helical proteins. The morphology of the nanofibers is composed of a uniform circular cross-section based on the images obtained using AFM, TEM, and SEM. The extracted nanofibers were successfully fabricated into transparent sheets that can be used in several applications.

## Abstract 2

We report the extraction of keratin nanofibers from the medulla of a parent yarn after denaturing the cuticle and cortex microstructures of a merino wool yarn.

## Graphical abstract

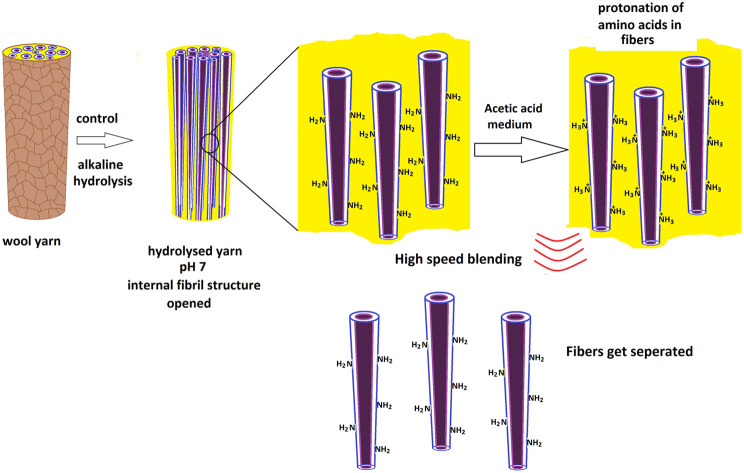

## 1. Introduction

Wool keratin, a fibrous cross-linked protein with high molecular weight, high cysteine content, and helical configuration, has attracted the attention of many researchers owing to its biocompatibility, non-toxicity, biodegradability, and natural abundance.^1–5^ An individual wool fiber consists of a bundle of α-helical keratin nanofiber filament core and proto filaments covered by an amorphous β-keratin protein matrix and overlapping cuticle cells, leading to its unique chemical and physical properties.^6–8^ These protofilaments have nonhelical N- and C-termini that are rich in cysteine residues and cross link with the wool matrix.^9^ Wool keratin consists of a highly conserved 19 amino acid sequence, rich in carbon, hydrogen, nitrogen, and sulfur linked together by peptide bonds to form ladder-like polypeptide chains.^10–12^ Thus, the scope of application of keratin nanostructures is wide and ranges from tissue engineering, wound dressing, target drug delivery vehicles, and plant fertilizers to water filtration, *etc.*^13–19^

Inspired by the self-assembly nature of cysteine-containing keratin proteins, many researchers have adopted a bottom-up approach to prepare protein nanofibers. In such studies, alkaline, enzymatic and acid hydrolyses of keratin wool or feathers or dissolution of the same in ionic liquids have resulted in de-crosslinking and disentanglement of protein molecules and finally regeneration in the form of nanofibers.^20–24^ Microwave induction, which is another method, has been explored for the green and efficient extraction of keratin from wool waste.^25^ Keratin has been extracted *via* sulphitolysis, a green method (no harmful chemicals), and processed into nanofibers from its solutions through electrospinning. Electrospinning is a versatile and easy-to-use technique for generating nanofibers. Additionally, many researchers have reported the preparation of keratin nanofiber composites, where the assembly of nanofiber architecture in the composite was performed through electrospinning or wet spinning of a keratin protein containing solution extracted from a natural source.^26–32^ Although these methods are efficient, they pose challenges, such as de-crosslinking and disentanglement of protein molecules, necessitating regeneration.

Many researchers have used a top-down approach to disintegrate biological nanofibers, such as chitin and cellulose, from their parent structure.^33–36^ Importantly, there is a notable gap in the literature concerning the extraction of keratin nanofibers by disassembling the hierarchical microstructure of wool fibers. To the best of our knowledge, there is no literature on extracting keratin nanofibers through disassembling wool fiber's hierarchical microstructure. The top-down method, which involves controlled alkaline hydrolysis and high-speed blending, offers potential for scalability, making it practical for future applications in large-scale production. For the first time, we report the extraction of α-helical keratin protein nanofibers from wool yarn through controlled alkaline hydrolysis, followed by high-speed blending in an acetic acid medium. Alkaline hydrolysis leads to hydrolyzing the β-sheet protein matrix of the cuticle and cortex that loosens the nanofiber filaments in the medulla of the wool fiber structure. A high-speed blender was used to disintegrate these loose protein nanofiber filaments from their parent structure in an acetic acid medium.

## 2. Materials and methods

### 2.1 Materials and chemicals

A merino wool yarn (∼15 μm diameter), glacial acetic acid (99% purity) and sodium hydroxide with a 99.8% purity level was used. An artificial snakeskin membrane with a 4000 MW cutoff was used for the dialysis of alkaline wool dispersion. All chemicals and materials were obtained from Sigma Aldrich.

### 2.2 Preparation of wool fibers

To remove the cuticles and cortex, the wool yarn samples were hydrolyzed in an alkaline medium. NaOH concentration and hydrolysis time were carefully controlled to avoid adversely hydrolyzing the inner fibrils of the wool yarn. For this, merino wool yarn was first washed using distilled water to remove dust particles or impurities. A weight of 0.5 g of wool yarn was hydrolyzed in 40 ml of 1 M, 2 M, 3 M, 4 M and 5 M NaOH solutions for 10 minutes at room temperature. Wool in alkaline dispersion was dialyzed using an artificial snakeskin membrane having 4000 MW cutoff, with distilled water until the pH of the wool dispersion became neutral. The dialysis allows the removal of the remaining NaOH, dissolved chemicals during hydrolysis and low molecular weight polypeptides (MW < 4000) obtained during the hydrolysis process. The hydrolyzed wool samples, after dialysis, are at a neutral pH (pH 7). A neutral pH condition is necessary for the hydrolyzed wool dispersant to further process and extract the nanofibers. The label given in 1 M wool hereinafter represents wool fibers hydrolyzed using 1 M NaOH and dialyzed. Similarly, the labels 2 M wool, 3 M wool, 4 M wool, and 5 M wool represent the product obtained after hydrolysis in the given concentration of NaOH and dialysis.

### 2.3 Extraction of wool nanofibers from prepared wool fibers

**Fig. 1:**
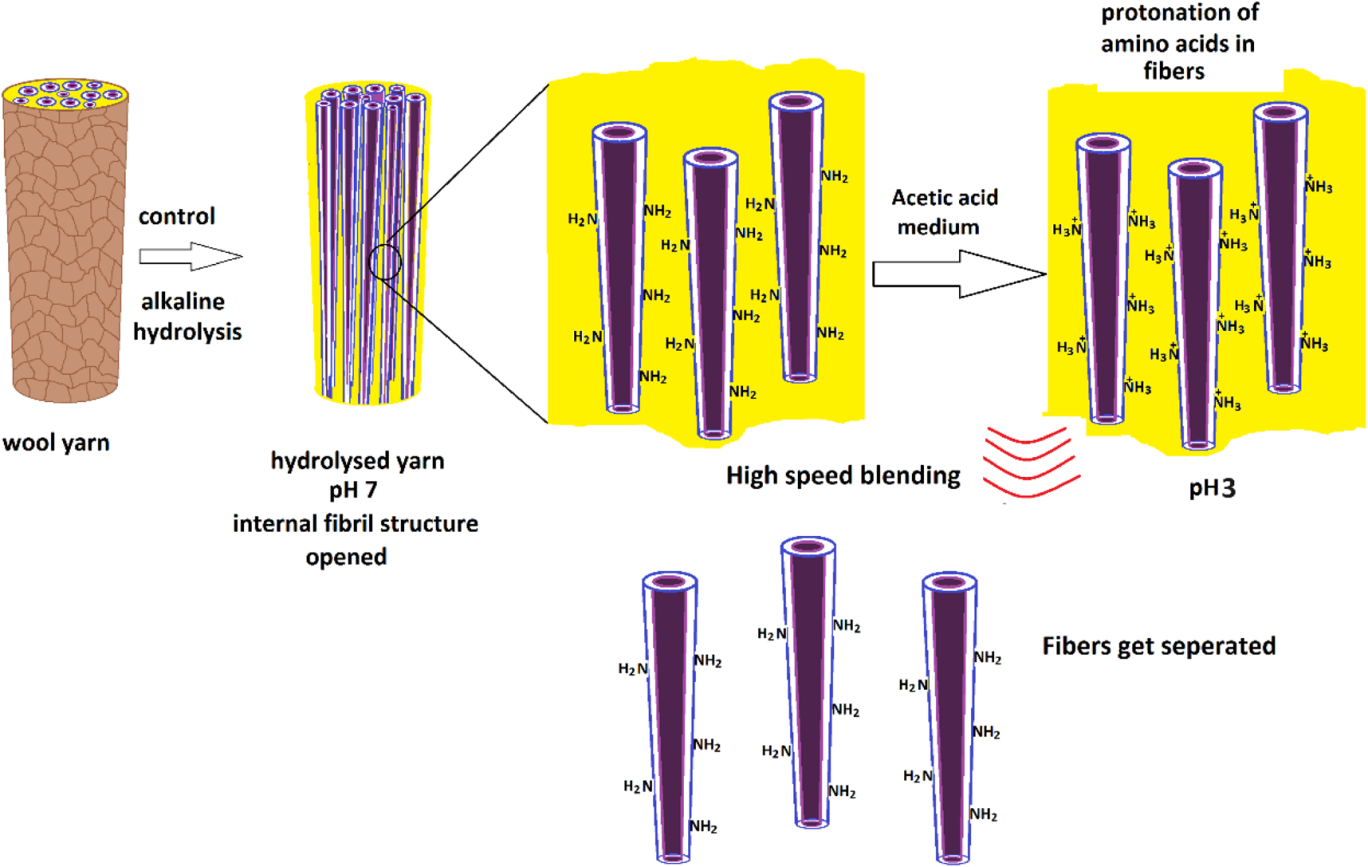
Schematic presentation of experimental flow for the preparation of protein nanofibers from merino wool fibers.

1 ml of glacial acetic acid was diluted in 100 ml of water to prepare a 1% acetic acid solution. Hydrolyzed wool yarn (0.015 g) was dispersed in 30 ml of 1% acetic acid solution. The wool dispersion was subjected to agitation using a high-speed blender, Innovex, 130 W, operated at 3000 rpm for 10 minutes at ambient temperature. Fig. 1 shows the experimental flow of the nanofiber preparation.

## 3. Characterization

The morphologies of wool yarn, alkaline hydrolyzed wool yarn, and nanofibers extracted from hydrolyzed wool yarn were studied using the Atomic Force Microscopic (AFM) Park system. AFM images were obtained using an XE-100 microscope. The measurements were performed in air at room temperature using non-contact mode with Si tips of the 1650–00 type scanning at a rate of 0.5 Hz. The wool yarn was firmly fixed to the AFM sample stub using double scotch tape for the analysis.

The extracted nanofibers were dispersed in an aqueous medium. A drop of the prepared solution was placed on a synthetic mica sheet, which acted as an atomically smooth support. Water was allowed to evaporate at room temperature before analysis. The mica sheet was firmly fixed to the AFM sample stub using double scotch tape for the analysis.

The morphology of the surface of wool fiber and yarns was characterized using a Hitachi SU6600 Schottky field emission scanning electron microscope (FE-SEM) operated at 15 kV accelerated voltage.

Fourier Transform Infrared spectra (FT-IR) of wool yarn and protein nano fibers in the dried solid state were obtained using Bruker, Vertex 80. Attenuated Total Reflectance (ATR) measurement mode was used with the sample mounted on a diamond crystal. Absorption spectra were recorded in 256 scans ranging from 800 cm^−1^ to 4000 cm^−1^ with 1 cm^−1^ resolution.

Thermo Gravimetric Analysis (TGA) was performed for wool yarns and protein nano fibers in the dried solid state using SDTQ-600 TGA from TA Instruments. Samples were heated from 30 °C to 1000 °C at a heating rate of 20 °C min^−1^ under nitrogen atmosphere. The nitrogen gas purge flow rate was set at 100 ml min^−1^.

DSC was performed for wool fibers under an inert environment at a ramp of 5 °C min^−1^ from room temperature to 500 °C using Q-200 DSC TA Instruments.

X-Ray Photoelectron Spectroscopic (XPS) measurements were carried out using EXCALABXi^+^ hemispherical electron analyzer (Thermo Fisher Scientific, United Kingdom) with monochromatic Al Kα radiation (*hν* = 1486.6 eV), and Thermo Avantage (5.982) software was used to perform curve fitting and to calculate other XPS parameters. Prior to the analysis, wool yarn and wool yarn dispersions were drop cast on a glass substrate and allowed to dry.

Nanofiber morphology was also characterized using a Transmission Electron Microscope (TEM) JEOL JEM-2100, operating at an accelerating voltage of 100 kV. Aqueous dispersions of the protein nanofibers were drop cast on a holey carbon copper grid and allowed to dry in the air prior to analysis.

## 4. Results and discussion

**Fig. 2:**
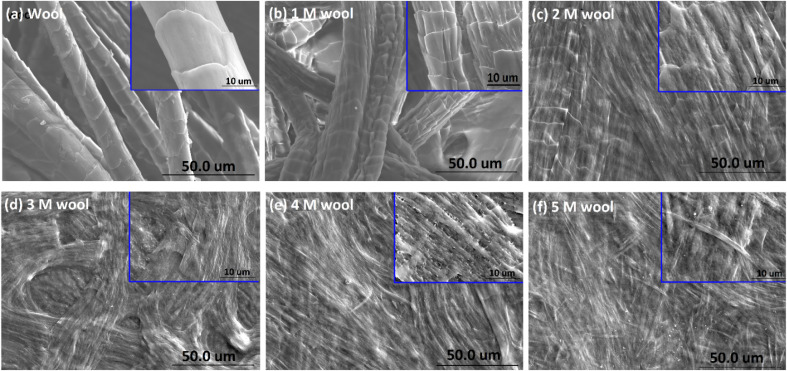
SEM micrograph of (a) wool yarn, (b) 1 M wool, (c) 2 M wool, (d) 3 M wool, (e) 4 M wool and (f) 5 M wool.

The surface morphological features of wool yarn and alkaline hydrolyzed wool yarn (with different initial alkaline concentrations) are shown in Fig. 2.

Fig. 2(c–f) shows the SEM images of the product obtained by hydrolyzing wool fibers with the given concentration of NaOH and dialyzing. The inset of each SEM micrograph shows a magnified image of the hydrolyzed wool sample.

As shown in Fig. 2, the pristine wool yarn surface consists of a scale structure comprising cuticle cells. These cuticle cells of the hierarchical microstructure of wool yarn sheath the cortex and medulla. The cuticle cells are overlapped, and the exposed edges face in one direction along the length of the yarn (Fig. 2(a)). The surface of the individual cells was observed to have a smooth structure. After being subjected to alkaline hydrolysis, the yarn surface showed denaturing of its outermost layer (Fig. 2). With the increase in the alkaline concentration of NaOH (from 1 M to 5 M) during hydrolysis, the total removal of these cuticle cells was observed. According to the SEM study, it is clear that the cuticle cells of the wool yarn were removed, and the cortex, which is the second hierarchical structure of the wool yarn, was exposed to the 4 M wool and 5 M wool.

**Fig. 3:**
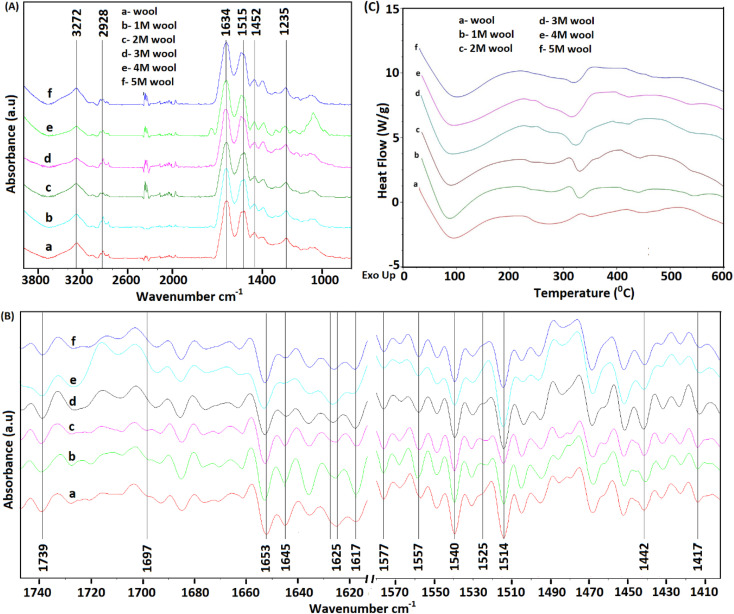
(A) FT-IR spectra (4000–800 cm^−1^) of pristine wool yarn and alkaline hydrolyzed wool yarn. (B) Second derivative infrared spectra of pristine wool yarn and alkaline hydrolyzed wool yarn. (C) DSC analysis of the pristine wool yarn and alkaline hydrolyzed wool yarn samples.

As depicted in Fig. 3A, FT-IR spectra of pristine wool yarn and alkaline hydrolyzed wool yarn samples showed an absorption band in the wavelength range of 4000–800 cm^−1^. These absorption bands correspond to the stretching vibration frequency of N–H connected with Amide A (3272 cm^−1^) and Amide B (3065 cm^−1^) in the wool samples. These bands are influenced by the Fermi resonance between the first overtones of Amide II.^37^ For pristine wool and surface-hydrolyzed wool, the band at 2926 cm^−1^ is related to the stretching vibration mode of the C–H bonds present in the samples. The mid-infrared spectrum with keratin absorption bands at 1629 cm^−1^, 1515 cm^−1^ and 1250 cm^−1^ can be designated as Amide I, Amide II and Amide III, respectively.^38^ These bands can be used to study the conformational arrangement of the protein molecules in wool samples. More complex vibration modes can be derived from in-plane bending of N–H, C–N stretching, C–C stretching and C–O bending in the Amide III band.^4,39^ Owing to the complex vibration modes of the Amide III band, it is not easy to identify the correlation between the Amide III band shape and the protein secondary structure. Therefore, the Amide I and Amide II bands are used to extract information about the secondary structure of the protein presence in wool.^40^ The absorption band for Amide I is mainly due to the stretching vibration of C=O. The absorption band for Amide II is derived primarily from the in-plane bending of N–H. This band also has minor contributions from C–N and C–C stretching vibrations.

To extract information about the secondary structure of the protein presence in wool, the broad Amide I and Amide II bands were resolved using the second derivative of the FT-IR spectrum of the wool samples. The corresponding spectra are given in Fig. 3B. It is worth noting that the peak positions are inverted in the second-order derivative spectra.

As depicted in Fig. 3B, the band at 1740 cm^−1^ corresponds to the C=O stretching vibration of free carboxyl groups, which belong to the glutamic and aspartic acids of wool protein.^20^ For the pristine wool sample (see Fig. 3B(a)), a broader peak was observed in the absorption band at 1625 cm^−1^ due to the presence of Amide I in the randomly arranged β-sheet structure.^41^ A significant reduction in the absorption intensity and narrowing of the peak was observed as the strength of the alkaline hydrolysis of the sample increased (Fig. 3B(b–f)). This indicates the chemical denaturing of the β-sheet present in the pristine wool due to alkaline hydrolysis. The peak at 1617 cm^−1^ represents the Amide I group present in β-sheet structure, which is bound with excess water molecules.^37^ Compared with the pristine wool sample (Fig. 3B(a)), 1 M, 2 M and 3 M wool samples (Fig. 3B(b–d)) showed an increase in the absorption intensity relative to the absorption peak intensity at 1625 cm^−1^, indicating that the sample has high surface bound water compared to that of the pristine wool sample. Contradictorily, the same absorption peak in the 4 M and 5 M wool yarn samples showed a reduction in peak intensity relative to the absorption peak intensity at 1625 cm^−1^, indicating that the 4 M and 5 M wool samples have low surface bound water compared to the pristine wool sample. The absorption band at 1653 cm^−1^ corresponds to the Amide I group present in the α-helix protein, which is the characteristic of the intermediate filament structure of the pristine wool yarn.^5^ According to Fig. 3(B), there was no observable reduction in the intensity of this peak with the increase in hydrolysis conditions. Therefore, this confirms that the α-helix structure of the protein is present in each hydrolyzed wool sample (1–5 M wool samples). The peak at 1645 cm^−1^ represents Amide I groups present in α-helix structure, which is bound with excess water molecules. Compared with the pristine wool sample (Fig. 3B(a)), all 1 M to 5 M wool samples (Fig. 3B(b–f)) showed a reduction in the absorption intensity relative to the absorption peak intensity at 1653 cm^−1^, indicating that the specific sample has a low amount of water bound α-helix structures compared to that of the pristine wool sample.

A weak absorption band was observed at 1697 cm^−1^ for the pristine wool sample (Fig. 3B(a)). This band corresponds to the disordered keratin conformations present in the β-sheet.^41^ β-Sheets are the typical constituent of the cuticle cells (outer layer of the wool yarn), and this matrix covers the intermediate filament structure of the wool yarn.^5^ Compared with the pristine wool yarn (Fig. 3B(a)), there is a significant decrease in the absorption bands at 1697 cm^−1^ (Fig. 3B(b–f)) for alkaline hydrolyzed wool yarn. This further confirms the gradual removal of β-sheet structure (cuticle cells) of the wool yarn with the increase in hydrolysis conditions.

For the wool samples, the shoulder peak at 1578 cm^−1^ (Fig. 3B(a–f)) is attributed to the asymmetric COO^−^ stretching vibrations, which come from aspartic and glutamic acid side chains.^42^ The absorption band at 1557 cm^−1^ corresponds to the Amide II present in the α-helix structure of the pristine wool and alkaline hydrolyze wool samples. Additionally, the Amide II groups present in β-sheet structure give an absorption band at 1531 cm^−1^ for the pristine wool sample (Fig. 3B(a)). This peak shows a gradual decrease in the peak intensity with the increase in alkaline hydrolysis of the wool sample (Fig. 3B(b–f)). This provides evidence for the denaturing of β-sheet structure that sheaths the α-helix intermediate filament structure of the wool yarn. The strong peaks at 1514 cm^−1^ and 1541 cm^−1^ (Fig. 3B(a–f)) correspond to the parallel and perpendicular Amide II groups of the α-helix protein presence in wool samples.^37^ In addition, the decoupled C–N stretching vibrations of the Amide II group were observed at 1437 cm^−1^ and 1418 cm^−1^ (Fig. 2B(a–f)).

The information obtained using FT-IR confirmed that the β-sheet, which sheaths the intermediate filament structure of the wool yarn, can be removed by subjecting the wool yarn sample to alkaline hydrolysis. As the controlled alkaline hydrolysis first denatures the β-sheet of the wool, α-helix protein that characterizes the intermediate filament structure of the yarn is intact and present on the sample. Therefore, controlled alkaline hydrolysis allows the intermediate filament structure of the wool yarn to be loosened for further processing.

Fig. 3(C) shows the DSC thermograms of wool, and alkaline hydrolyzed wool samples. A large endothermic peak was observed at around 90 °C owing to the evaporation of water from the wool samples. The area of this peak showed an increase for 1 M, 2 M and 3 M hydrolyzed wool samples (Fig. 3C(b–d)). This can be due to the presence of more moisture in β-sheet structures of the alkaline hydrolyzed wool sample compared with the control wool sample and 4 M and 5 M hydrolyzed wool samples (Fig. 3C(e and f)), which was further observed in the FTIR study. Additionally, two endothermic peaks were observed for the wool samples at the temperature range of 230–350 °C. These peaks correspond to the thermal denature of amorphous and crytstalline α form and β-sheet matrix structure of wool proteins.^43^ The bimodal endothermic peak at the temperature range 240–280 °C was observed in the DSC thermogram of the pristine wool sample (Fig. 3C(a)). Here, the peak at 240 °C corresponds to the thermal decomposition of the amorphous α form, which is mainly the cystine rich matrix in the wool sample. This peak is overlapped by the degradation of other chemical components, which include denaturation of other histological components^43^ and are observed around 270 °C. It is noteworthy that with the increase in the hydrolysis conditions, there is an observable reduction in the endothermic peak at 240–280 °C (Fig. 3C(b–f)), which can be attributed to some extent to the denature of cystine rich matrix structure, with a degradation temperature around 240–280 °C. Therefore, alkaline hydrolysis effectively interacted with the amorphous matrix of the wool sample, with lower resistance to the chemical reaction. Moreover, compared to the pristine wool sample, the endothermic peak at 350 °C (Fig. 3C(a)) showed a shift to the lower temperature range at 330 °C in alkaline hydrolysed wool samples (Fig. 3C(b–f)). For 1 M, 2 M and 3 M wool samples, the area under the endothermic peak is higher compared to the pristine wool. The thermogram of the 4 M and 5 M wool samples showed a reduction in the area under the endothermic peak at 330 °C compared with the other hydrolyzed wool samples. However, there was an increase compared with the pristine wool sample. The increase in the peak area can be explained by the fact that water molecules present in the hydrolyzed wool (which is also evidenced from the FTIR study) sample interact with the polypeptide when the crystallites of the sample melt at higher temperatures. An increase in the peak area is attributed to an increase in the α-cystallities of the hydrolysed wool sample compared to the pristine wool sample.^44^ Therefore, for the 4 M and 5 M wool samples although there was no evidence for the bound water in its structure, the thermogram showed a comparable high peak area under the endothermic peak at 330 °C due to the increased amount of α-cystallities compared to the pristine wool sample.

**Fig. 4:**
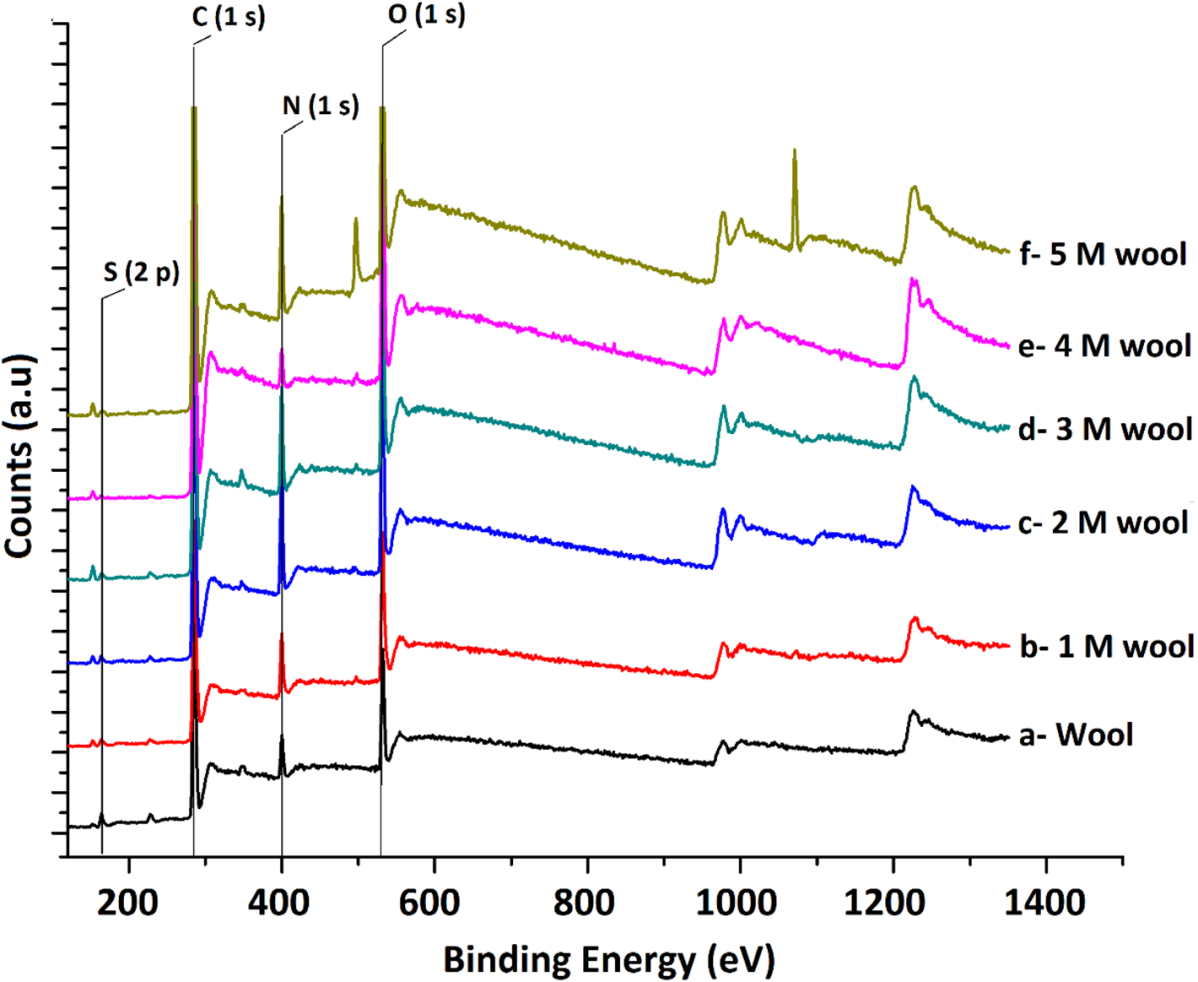
XPS spectra of pristine wool and alkaline hydrolyzed wool samples.

The XPS surface analysis technique was used to gather information about the elemental composition of wool and hydrolysed wool yarn samples in their immediate subsurface up to a depth of about 3 nm. Wide scan XPS spectra of the wool samples and alkaline hydrolysed wool samples showed the characteristic lines of carbon, nitrogen, oxygen and sulfur. For these samples, C (1s), N (1s), O (1s) and S (2p) photo electron peaks were observed at 284.75, 399.95, 531.75 and 164.5 eV, respectively (Fig. 4(a–f)). Similarly, the hydrolysed wool samples obtained under different alkaline hydrolysis conditions showed spectra comparable to those of the pristine wool sample. However, there was an apparent difference in the relative photo electron line intensities.

From the photoelectron line intensities obtained using XPS spectra, the surface element ratios were calculated, as shown in Table 1.

**Table 1:** Surface elemental ratio of wool and hydrolyzed wool obtained *via* XPS analysis

Sample	Relative atomic ratio				N/S ratio
	C	O	N	S	
Wool	1	0.13	0.076	0.023	3.30
1 M wool	1	0.25	0.128	0.023	5.56
2 M wool	1	0.25	0.160	0.016	10.00
3 M wool	1	0.27	0.195	0.011	17.73
4 M wool	1	0.23	0.136	0.011	12.36
5 M wool	1	0.184	0.036	0.002	18

The results show that the N/C and O/C ratios of the pristine wool are lower than those of the hydrolysed wool yarn samples. This can be attributed to the presence of carbon-rich lipids in the external membrane (epicuticle) that surrounds each cuticle cell of the pristine wool sample.^45^ With the increase in alkaline hydrolysis conditions, an increase in the N/C and O/C ratios was observed. Higher surface N/C and O/C ratios in hydrolyzed wool yarn samples suggest that there is a small lipid layer and that there is a lesser amount of cuticle cells present in exposed surface layers of 1 M, 2 M, 3 M, 4 M and 5 M wool samples. This result also agrees with the FT-IR and DSC results discussed above.

Sulfur analysis of the wool sample is important in this study. In contrast to N/C and O/C ratios, the S/C ratio showed a gradual decrease from the pristine wool sample to the 4 M hydrolysed wool sample. For the 5 M wool sample, the S/C ratio showed a slight increase compared to the 4 M wool sample. Sulfur is a main constituent of the cuticle cell (high sulfur matrix). The N/S ratio of a cuticle cell free cortical intercellular structural components is expected to be 13. A lower value than this is expected if the XP emission from the cortex of the wool sample arises from a combination of both high sulfur (matrix) and low sulfur protein (fibrillar structure).^46^ Therefore, for the 2 M wool sample with an N/S ratio of 10, it can be concluded that the XPS signals are from a surface where a trace amount of cuticle cells is present on the surface. This observation is also supported by the SEM images of the 2 M wool sample. The N/S ratio of the 3 M wool sample was calculated as 17.7, indicating that the removal of the cuticle cells and XPS signals is from the intracellular structural components of the sample. For the 4 M wool sample, this value is reduced to 12.36, indicating that the XPS signals are from the cortex of the wool samples, which includes a high sulfur matrix. As the hydrolysis condition increases, the N/S ratio of the 5 M wool samples increases to 18, which indicates that the removal of the matrix structure of the cortex and signals is only from the low sulfur baring fibrillar structure of the wool sample. Therefore, it can be confirmed from the XPS results that the alkaline hydrolysis of wool yarn successfully removed the epicuticle, cuticle cells, and cortex from its structure and that the internal fibrillar surface was exposed in a 5 M wool sample.

**Fig. 5:**
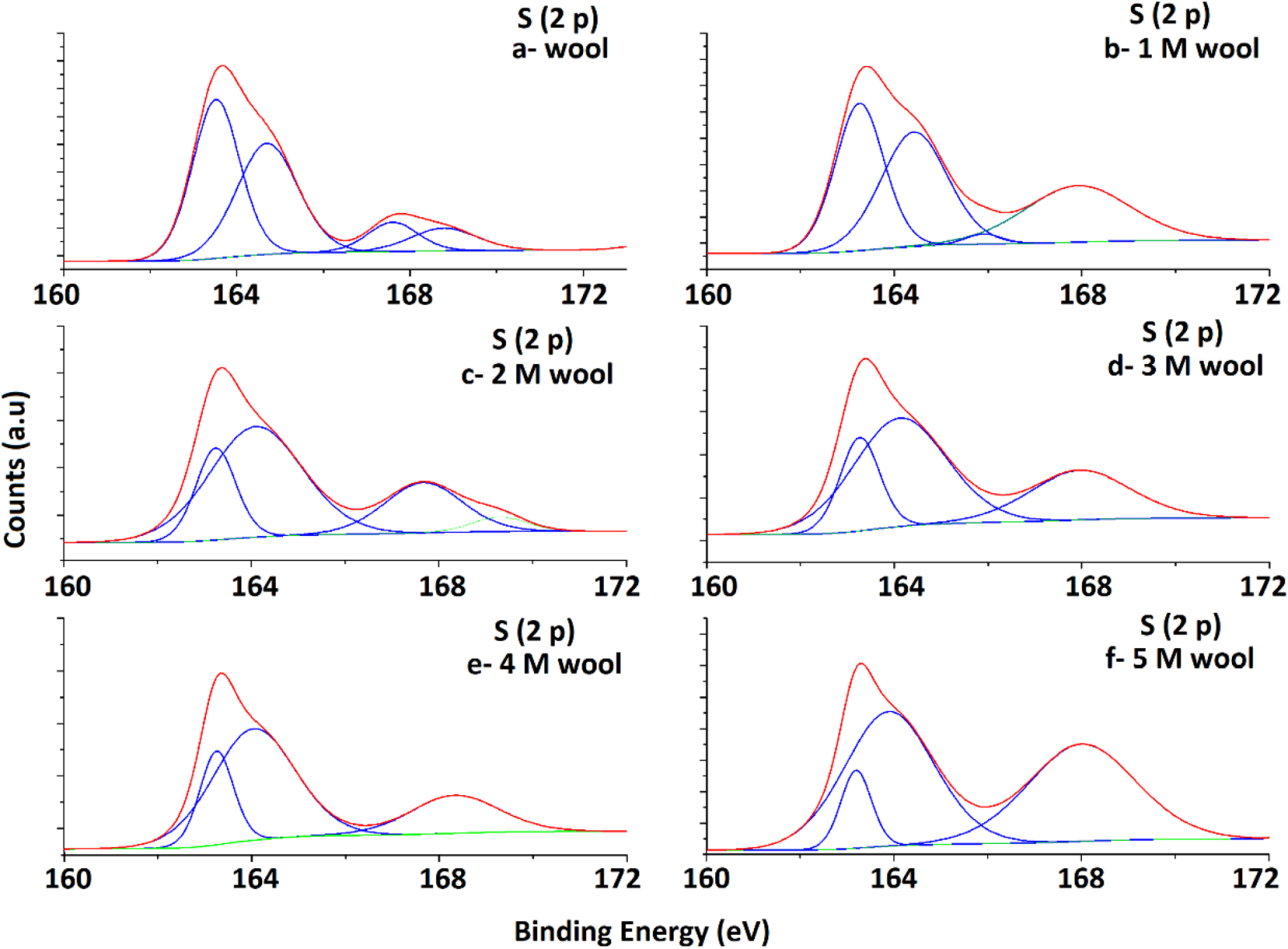
Deconvoluted S 2p spectra of pristine wool and alkaline hydrolysed wool samples obtained through XPS analysis.

High-resolution S 2p spectra obtained for pristine and alkaline hydrolyzed wool samples are deconvoluted to their Gaussian peaks, as illustrated in Fig. 5. S 2p spectra mainly consist of a major broad peak at 164.5 eV due to the presence of native disulfide (S^2−^) bonds of cystine residues^47^ and a minor peak at 168 eV attributable to the oxidized (S^6+^) species.^50^ For the pristine wool sample, the intensity of the peak at 164.5 eV is relatively higher compared to the peak at 168 eV (Fig. 5(a)), which indicates that the sample contains more disulphide bonds in its structure. With the increase in hydrolysis conditions, the peak intensity at 164.5 eV gradually decreased and the peak intensity at 168 eV increased. This clearly shows the oxidation of S-baring compounds, which are mainly the cystin residues in the wool yarn structure due to alkaline hydrolysis. This observation is also in accordance with the observations obtained in the DSC analysis. The broad peak at 164.5 eV consists of two individual Gaussian curves approximately at 163.5 eV and 165 eV. The peak at 165 eV is mainly due to the presence of sulfur in the intermediate (4+) oxidation state.^48^ Compared with the spectra of the pristine wool sample (Fig. 5(a)), the hydrolyzed wool sample showed a gradual decrease in the Gaussian peak at 163.8 eV and an increase in the peak intensity of the Gaussian peak at 165 eV for 1–5 M wool samples (Fig. 5(b–f)). Therefore, it can be concluded that disulphide bonds are converted into their intermediate oxidation state through alkaline hydrolysis. Furthermore, for the peak at 165 eV, the intensity of the peak at 168 eV showed an increment for 1–5 M wool samples (Fig. 5(b–f)). This observation is attributed to the loss of the intermediate oxidation state of the sulphur baring groups with an increase in the hydrolysis level of the wool sample. Alkali attacks on wool peptide linkages contribute to the degradation of amino acids, such as arginine, serine, threonine, and cystine. Alkaline hydrolysis primarily attacks the asparagine and glutamine (amide bond) and breaks peptide bonds in the major chains, resulting in amino acid racemization.^49^

According to the above studies, it was identified that the cuticle cells and cortex denatured in a 5 M wool sample. Under 5 M hydrolysis conditions, the internal fibrous structure of the wool yarn was loosened, and the external surface was exposed. Once the external sheath is removed, these fiber bundles remain in compact form. Additionally, it was identified by subjecting these fiber bundles to ultrasonication, resulting in fragments of fibers and other heterogeneous structures (ESI, Fig. S1†). Therefore, the mechanical treatment of the hydrolysed wool sample needs to be controlled carefully to extract nano fibers from the wool yarn.

Therefore, it is assumed that the energy given during ultrasonication will denature the protein, and this is not the most suitable method for the extraction of protein nano fibers. Therefore, to further loosen these fibrous structures from the 5 M wool sample, the first fibers were given a surface charge by dispersing the sample in an acetic acid medium. In the presence of acetic acid, free amine groups in a 5 M wool sample were assumed to protonate. It is hypothesized these positive charges allow the wool fiber bundles to separate into individual nano fibers in the presence of high mechanical force during high seed blending.

**Fig. 6:**
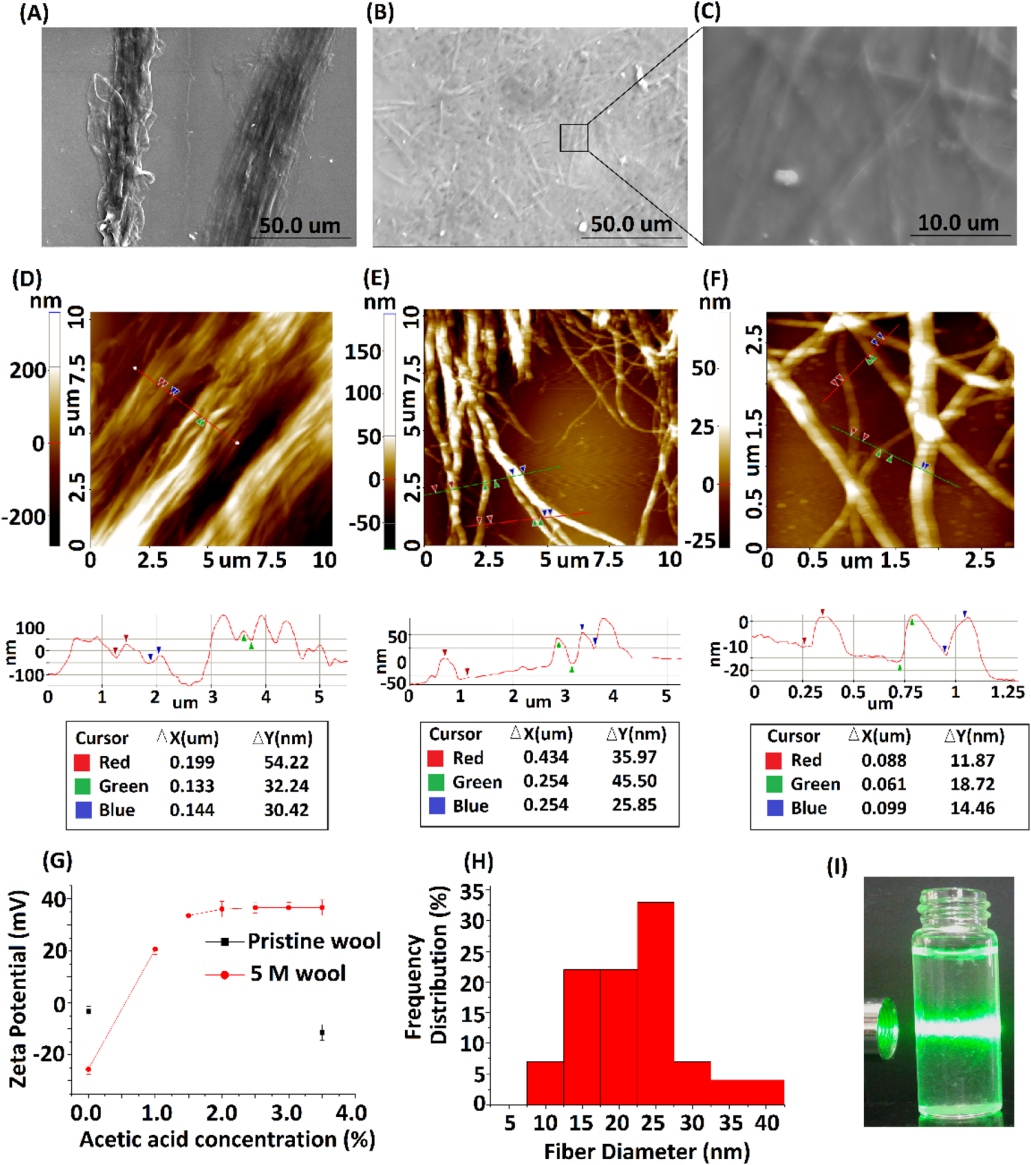
(A) SEM micrograph of 5 M wool sample dispersed in 1.5% acetic acid medium, (B) SEM micrograph of the extracted protein nano fibers, (C) magnified SEM image of protein nano fibers, (D) AFM topographical image of the 5 M wool sample dispersed in 1.5% acetic acid medium, (E) AFM topographical image of protein nano fibers, (F) magnified AFM image of protein nano fibers, (G) zeta potential measurement of the aqueous dispersion of pristine wool and 5 M wool samples in different acetic acid concentrations, (H) fiber diameter distribution of protein nano fibers and (I) laser light scattering on protein nano fiber dispersion.

The 5 M wool sample was dispersed in distilled water at 0.5 g L^−1^ concentration. To introduce a surface charge on these fiber bundles, acetic acid was added to the dispersion at different concentrations. The zeta potential of the material dispersion was measured, as illustrated in Fig. 6G. For the pristine wool yarn sample and surface-hydrolyzed wool yarn sample, the zeta potential remained at −3 mV and −25 mV, respectively, in the absence of acetic acid in the wool fiber dispersion. The surface charge of pristine wool yarn remained unchanged at −10 mV in the presence of acetic acid. For the hydrolyzed wool yarn sample, it was observed to have +20 mV zeta potential at 1% acetic acid and gradually increased to a maximum surface charge of +36 mV at 3.5% acetic acid. The results show that the positively charged groups are predominant on the surface of the hydrolyzed wool fiber in an acetic acid medium. The SEM micrograph and AFM surface topographic images of the 5 M wool yarn sample in a 1.5% acetic acid medium are shown in Fig. 6A and D, respectively. The SEM micrograph shows that the sample contains individual fibers arranged parallel to the length of the wool fiber. AFM topographical image (Fig. 6D) revealed characteristic parallel ridges and groves on the sample surface, which is due to the presence of nano fibers. The surface scan line profile of this image shows that these ridges and groves have an average height of 34 nm.

To further separate these protein fibers into their nanofibers, a 5 M wool sample in a 1.5% acetic acid medium was subjected to high-speed blending. The morphologies of the samples were analyzed. SEM and AFM micrographs depicted in Fig. 6B, C, E and F show the morphological transition of the 5 M wool sample to individual wool nanofibers during high-speed blending in an acetic acid medium. SEM micrographs (Fig. 6B) show the presence of disintegrated fibers in the sample. The magnified image shows that these fibers are assembled in a non-ordered array (Fig. 6D).

AFM topographical image (Fig. 6E) shows that the sample consists of randomly oriented nanofibers. The surface line scan profile of this topographical image shows a fiber diameter of ∼34 nm (Fig. 6E line profile scan) and has a uniform cross-section along the length of the fiber. The magnified AFM topographical image (Fig. 6F) of the fibers clearly shows that the sample consists of protofibrils with a tubular morphology and smaller fibers that have individual diameters of around 12 nm. The histogram of fiber diameter and length distribution illustrated in Fig. 6H was obtained by analyzing three AFM topographical images of the sample (ESI, Fig. S2†). The average size of nano fibers obtained from the sample is 25 nm in diameter and ∼3 μm in length. Insert image of the protein nano fiber dispersion in an aqueous medium (Fig. 6I) shows the scattering of laser light due to the presence of nano fibers in the dispersion medium.

**Fig. 7:**
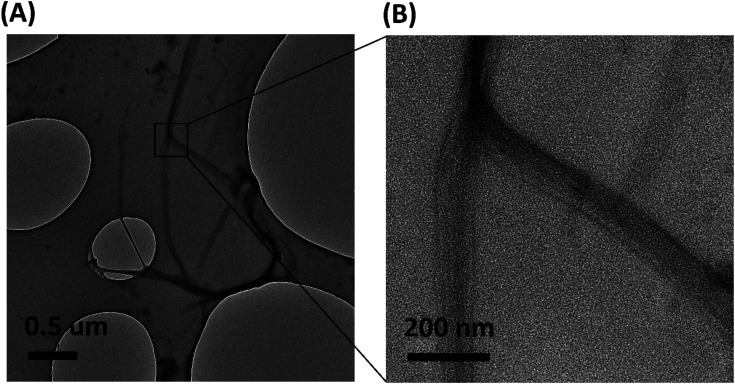
(A) TEM image of protein nano fibers extracted from 5 M wool sample and (B) magnified image of the protein nanofibers.

Transmission electron microscopic image of the sample shows the presence of protein nano fibers with a fiber length >2 μm in the dispersion medium. Most fibers have a diameter of <45 nm (ESI Fig. S3†). TEM images indicate that these fibers are in bundle form and amorphous in nature (Fig. 7B). TEM EDX analysis further confirms the presence of C, N and O (ESI Fig. S4†), confirming the presence of keratin protein nano fibers in the suspension.

**Fig. 8:**
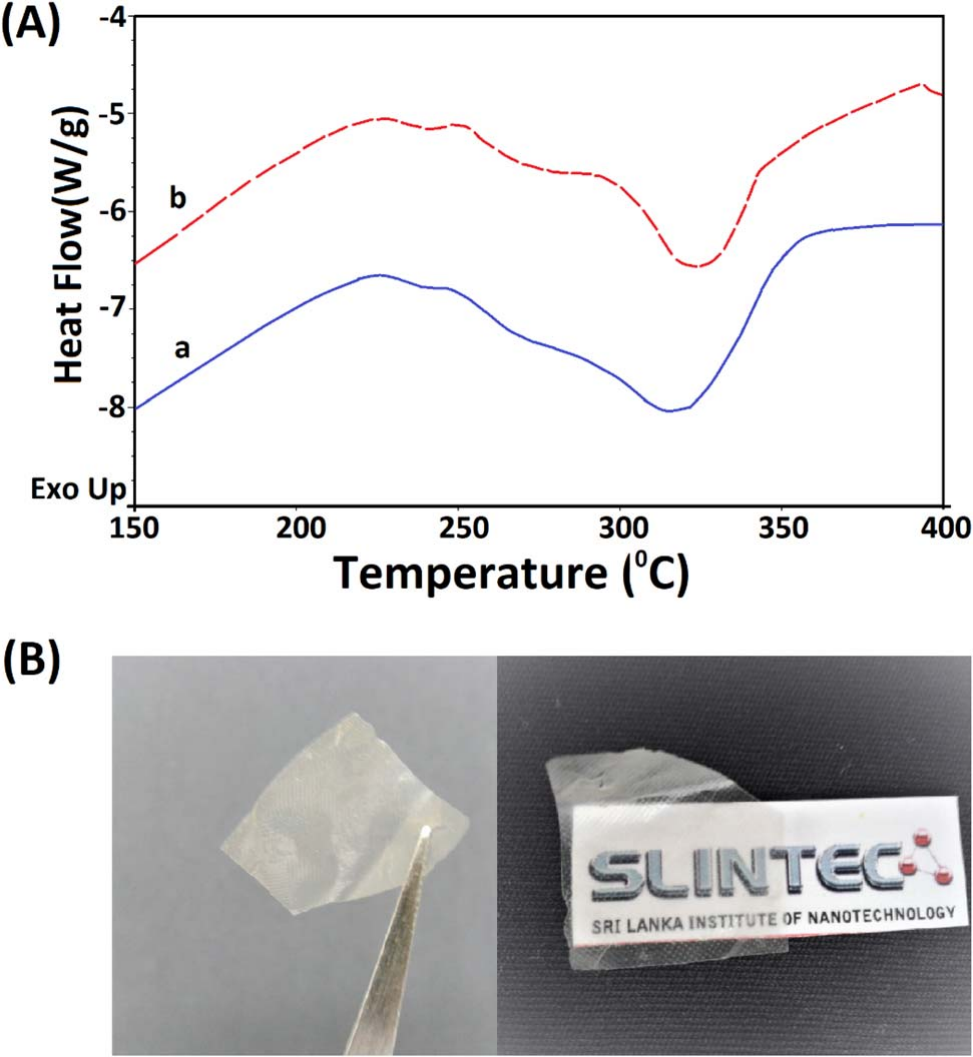
(A) TG analysis of the (a) 5 M alkaline hydrolyzed wool fibers and (b) extracted keratin protein nano fibers and (B) transparent thin film fabricated using the extracted keratin protein nano fibers.

The extracted nano fibers were further analysed using a thermo gravimetric analyzer. Fig. 8A(a and b) shows the thermograms of 5 M wool and extracted wool nano fiber. The two thermo grams give identical endothermic peaks, demonstrating that both samples have similar structures. In both samples, two endothermic peaks were observed in the temperature range 230–350 °C. As discussed in the thermal analysis of the hydrolysed wool yarn compared to the pristine wool sample, these peaks correspond to the thermal denature of amorphous and crytstalline form of α and β-sheet matrix structure of wool proteins.^43^ Additionally, a bimodal endothermic peak can be observed at the temperature range of 240–280 °C (Fig. 8A(a and b)). These peaks are low in intensity for both the 5 M wool (Fig. 8A(a)) and extracted wool nano fiber samples (Fig. 8A(b)). This confirms that alkaline hydrolysis results in the denature of cystine rich matrix structure, which has a degradation temperature of around 240–280 °C. The 5 M wool and extracted nano fiber sample showed an endothermic peak at 330 °C, which corresponds to the melting of α-cystallities in the structure.^44^ The intensities of the peaks are comparable. The thermal analysis shows that the extracted wool nano fibers are rich in α-helical protein. The use of both chemical and mechanical treatment has resulted in the extraction of α-helical protein nano fibers from pristine wool fibers using a top down approach.

The extracted nano fibers dispersion was cast to a Petridish and dried in a vacuum oven at 40 °C. A thin transparent sheet was obtained, as shown in Fig. 8B. α-Helical protein nano fibers extracted using the reported methodology can be applied to fabricate thin films. These keratin protein thin films can be used in many fields. Some studies have reported the use of keratin protein thin films in biomedical applications and as a biocompatible, high capacitance dielectric layer as well as a supporting substrate for organic thin film transistors.^50–52^

## 5. Conclusion

Merino wool yarn consists of α keratin protein nano fibers embedded inside its hierarchical architecture. A simple top down approach was used in this study to disintegrate these nano fibers from their parent structure. Cuticle cells, epicuticle and cortex of the wool can be dissolved in an alkaline medium. Under controlled conditions, during hydrolysis, the internal fibrous α keratin protein can be separated. These fibers are compacted in a bundle form and are likely to generate a positive charge on their surface in the presence of acetic acid in the dispersion medium. They dissolve and denature in highly concentrated acids, alkaline media or under high mechanical forces. Thus, in this study, we mainly focused on using a mild surface chemical modification method combined with mechanical agitation to disintegrate the fiber bundle into its fibers. Controlled hydrolysis of wool fibers in 5 M NaOH allowed the dissolution of the outermost layer of the fibers. Owing to the surface amine functional groups present in these hydrolyzed fibrous structures, a surface charge was introduced in a mild acid medium using 1% acetic acid. Intra-fiber repulsion due to these surface charges has allowed the compact fibrous structure, arranged parallel along the length of the wool fiber, to be loosened, as evidenced by the SEM and AFM analyses. Further, subjecting these loosed fibrous structures to mechanical force (high-speed blending) allowed them to disintegrate into individual nano fibers. Therefore, it can be concluded that a simple top down approach can be used to obtain α keratin protein nano fibers with a diameter of 25 nm and from merino wool yarn. The extracted α keratin protein nano fibers are fabricated into transparent thin films. These can be used for many applications, such as wound dressing, drug delivery, filtration processes, capacitors and thin film transistors.

## Conflicts of interest

There are no conflicts to declare.

## Supplementary Material

RA-014-D3RA07063H-s001

## References

[cit1] Steinert P. M. (1993). Structure, Function, and Dynamics of Keratin Intermediate Filaments. J. Invest. Dermatol..

[cit2] Magin T. M., Vijayaraj P., Leube R. E. (2007). Structural and Regulatory Functions of Keratins. Exp. Cell Res..

[cit3] Pan X., Hobbs R. P., Coulombe P. A. (2013). The Expanding Significance of Keratin Intermediate Filaments in Normal and Diseased Epithelia. Curr. Opin. Cell Biol..

[cit4] Wojciechowska E., Włochowicz A., Wesełucha-Birczyńska A. (1999). Application of Fourier-Transform Infrared and Raman Spectroscopy to Study Degradation of the Wool Fiber Keratin. J. Mol. Struct..

[cit5] Wojciechowska E., Rom M., Włochowicz A., Wysocki M., Wesełucha-Birczyńska A. (2004). The Use of Fourier Transform-Infrared (FTIR) and Raman Spectroscopy (FTR) for the Investigation of Structural Changes in Wool Fibre Keratin after Enzymatic Treatment. J. Mol. Struct..

[cit6] Zoccola M., Aluigi A., Vineis C., Tonin C., Ferrero F., Piacentino M. G. (2008). Study on Cast Membranes and Electrospun Nanofibers Made from Keratin/Fibroin Blends. Biomacromolecules.

[cit7] Idris A., Vijayaraghavan R., Rana U. A., Patti A. F., MacFarlane D. R. (2014). Dissolution and Regeneration of Wool Keratin in Ionic Liquids. Green Chem..

[cit8] Yu X. , Yang Y. and Xu H., Lightweight Materials from Biopolymers and Biofibers, ACS Symposium Series, American Chemical Society, 2014, 10.1021/bk-2014-1175

[cit9] McKittrick J., Chen P. Y., Bodde S. G., Yang W., Novitskaya E. E., Meyers M. A. (2012). The Structure, Functions, and Mechanical Properties of Keratin. JOM.

[cit10] Aluigi A., Tonetti C., Rombaldoni F., Puglia D., Fortunati E., Armentano I., Santulli C., Torre L., Kenny J. M. (2014). Keratins Extracted from Merino Wool and Brown Alpaca Fibres as Potential Fillers for PLLA-Based Biocomposites. J. Mater. Sci..

[cit11] Rouse J. G., Van Dyke M. E. (2010). A Review of Keratin-Based Biomaterials for Biomedical Applications. Materials.

[cit12] O'Cualain R. D. M., Sims P. F. G., Carr C. M. (2011). Structural Analysis of Alpha-Helical Proteins from Wool Using Cysteine Labelling and Mass Spectrometry. Int. J. Biol. Macromol..

[cit13] Khajavi R., Abbasipour M., Bahador A. (2016). Electrospun Biodegradable Nanofibers Scaffolds for Bone Tissue Engineering. J. Appl. Polym. Sci..

[cit14] Wang Y., Li P., Xiang P., Lu J., Yuan J., Shen J. (2016). Electrospun Polyurethane/Keratin/AgNP Biocomposite Mats for Biocompatible and Antibacterial Wound Dressings. J. Mater. Chem. B.

[cit15] Katoh K., Shibayama M., Tanabe T., Yamauchi K. (2004). Preparation and Properties of Keratin–Poly(Vinyl Alcohol) Blend Fiber. J. Appl. Polym. Sci..

[cit16] Aluigi A., Tonetti C., Vineis C., Tonin C., Mazzuchetti G. (2011). Adsorption of Copper(II) Ions by Keratin/PA6 Blend Nanofibres. Eur. Polym. J..

[cit17] Arshad M., Khosa M. A., Siddique T., Ullah A. (2016). Modified Biopolymers
as Sorbents for the Removal of Naphthenic Acids from Oil Sands Process Affected Water (OSPW). Chemosphere.

[cit18] Zhi X., Wang Y., Li P., Yuan J., Shen J. (2015). Preparation of Keratin/Chlorhexidine Complex Nanoparticles for Long-Term and Dual Stimuli-Responsive Release. RSC Adv..

[cit19] Tissera N. D., Wijesena R. N., Yasasri H., de Silva K. M. N., de Silva R. M. (2020). Fibrous Keratin Protein Bio Micro Structure for Efficient Removal of Hazardous Dye Waste from Water: Surface Charge Mediated Interfaces for Multiple Adsorption Desorption Cycles. Mater. Chem. Phys..

[cit20] Holkar C. R., Jadhav A. J., Bhavsar P. S., Kannan S., Pinjari D. V., Pandit A. B. (2016). Acoustic Cavitation Assisted Alkaline Hydrolysis of Wool Based Keratins To Produce Organic Amendment Fertilizers. ACS Sustain. Chem. Eng..

[cit21] Aluigi A., Zoccola M., Vineis C., Tonin C., Ferrero F., Canetti M. (2007). Study on the Structure and Properties of Wool Keratin Regenerated from Formic Acid. Int. J. Biol. Macromol..

[cit22] Poole A. J., Church J. S., Huson M. G. (2009). Environmentally Sustainable Fibers from Regenerated Protein. Biomacromolecules.

[cit23] Xu H., Yang Y. (2014). Controlled De-Cross-Linking and Disentanglement of Feather Keratin for Fiber Preparation via a Novel Process. ACS Sustain. Chem. Eng..

[cit24] Zheng S., Nie Y., Zhang S., Zhang X., Wang L. (2015). Highly Efficient Dissolution of Wool Keratin by Dimethylphosphate Ionic Liquids. ACS Sustain. Chem. Eng..

[cit25] Du W., Zhang L., Zhang C., Cao J., Wang D., Li H., Li W., Zeng J. (2022). Green and Highly Efficient Wool Keratin Extraction by Microwave Induction Method. Frontiers in Materials.

[cit26] Dickerson M. B., Sierra A. A., Bedford N. M., Lyon W. J., Gruner W. E., Mirau P. A., Naik R. R. (2013). Keratin-Based Antimicrobial Textiles, Films, and Nanofibers. J. Mater. Chem. B.

[cit27] Thompson Z. S., Rijal N. P., Jarvis D., Edwards A., Bhattarai N. (2016). Synthesis of Keratin-based Nanofiber for Biomedical Engineering. J. Visualized Exp..

[cit28] Yong L., Jia L., Jie F., Meng W. (2014). Preparation and Characterization of Electrospun Human Hair Keratin/Poly (Ethylene Oxide) Composite Nanofibers. Matéria.

[cit29] Fan J., Lei T.-D., Li J., Zhai P.-Y., Wang Y.-H., Cao F.-Y., Liu Y. (2016). High Protein Content Keratin/Poly (Ethylene Oxide) Nanofibers Crosslinked in Oxygen Atmosphere and Its Cell Culture. Mater. Des..

[cit30] Ma B., Qiao X., Hou X., Yang Y. (2016). Pure Keratin Membrane and Fibers from Chicken Feather. Int. J. Biol. Macromol..

[cit31] Aluigi A., Varesano A., Montarsolo A., Vineis C., Ferrero F., Mazzuchetti G., Tonin C. (2007). Electrospinning of Keratin/Poly(Ethylene Oxide)Blend Nanofibers. J. Appl. Polym. Sci..

[cit32] Babitha S., Rachita L., Karthikeyan K., Shoba E., Janani I., Poornima B., Purna Sai K. (2017). Electrospun Protein Nanofibers in Healthcare: A Review. Int. J. Pharm..

[cit33] Wijesena R. N., Tissera N., Kannangara Y. Y., Lin Y., Amaratunga G. A. J., de Silva K. M. N. (2015). A Method for Top down Preparation of Chitosan Nanoparticles and Nanofibers. Carbohydr. Polym..

[cit34] Lee K.-Y. , Tammelin T., Kiiskinen H., Samela J., Schlufter K. and Bismarck A., Nanofibrillated Cellulose vs. Bacterial Cellulose: Reinforcing Ability of Nanocellulose Obtained Top-down or Bottom-Up, in ECCM15-15th European Conference on Composite Materials, Venice, 2013

[cit35] Khalil H. P. S. A., Davoudpour Y., Saurabh C. K., Hossain M. S., Adnan A. S., Dungani R., Paridah M. T., Sarker M. Z. I., Fazita M. R. N., Syakir M. I. (2016). A Review on Nanocellulosic Fibres as New Material for Sustainable Packaging: Process and Applications. Renewable Sustainable Energy Rev..

[cit36] Wijesena R. N., Tissera N. D., Rathnayaka V. W. S. G., de Silva R. M., de Silva K. M. N. (2020). Colloidal Stability of Chitin Nanofibers in Aqueous Systems: Effect of PH, Ionic Strength, Temperature & Concentration. Carbohydr. Polym..

[cit37] Bendit E. G. (1966). Infrared Absorption Spectrum of Keratin. I. Spectra of α-, β-, and Supercontracted Keratin. Biopolymers.

[cit38] Miyazawa M. and Sonoyama M., Studies on the Structural Characterisation of Proteins M Second Derivative Near Infrared Studies on the Structural Characterisation of Proteins, 1998, vol. 6

[cit39] Gunasekera U. , Perera N., Perera S., Hareendra Y., Somaweera L., De Silva N., Tissera N. and Wijesinghe R., Modification of Thermal Conductivity of Cotton Fabric Using Graphene, in 2015 Moratuwa Engineering Research Conference (MERCon), IEEE, 2015, pp. 55–59, 10.1109/MERCon.2015.7112320

[cit40] Surewicz W. K., Mantsch H. H., Chapman D. (1993). Determination of Protein Secondary Structure by Fourier Transform Infrared Spectroscopy: A Critical Assessment. Biochemistry.

[cit41] Dong A., Huang P., Caughey W. S. (1990). Protein Secondary Structures in Water from Second-Derivative Amide I Infrared Spectra. Biochemistry.

[cit42] Wojciechowska E., Włochowicz A., Wysocki M., Pielesz A., Wesełucha-Birczyńska A. (2002). The Application of Fourier-Transform Infrared (FTIR) and Raman Spectroscopy (FTR) to the Evaluation of Structural Changes in Wool Fibre Keratin after Deuterium Exchange and Modification by the Orthosilicic Acid. J. Mol. Struct..

[cit43] Milczarek P., Zielinski M., Garcia M. L. (1992). The Mechanism and Stability of Thermal Transitions in Hair Keratin. Colloid Polym. Sci..

[cit44] Cao J., Joko K., Cook J. R. (1997). DSC Studies of the Melting Behavior of α-Form Crystallites in Wool Keratin. Text. Res. J..

[cit45] Dowling L. M., Jones L. N., Leaver I. H., Hughes A. E. (1988). TEM and X-Ray Photoelectron Spectroscopic Studies of Wool Fibers after Cuticle Removal. Text. Res. J..

[cit46] Carr C. M., Leaver I. H., Hughes A. E. (1986). X-Ray Photoelectron Spectroscopic Study of the Wool Fiber Surface. Text. Res. J..

[cit47] Millard M. M., Pavlath A. E. (1972). Surface Analysis of Wool Fibers and Fiber Coatings by X-Ray Photoelectron Spectroscopy. Text. Res. J..

[cit48] Bradley R. H., Clackson I. L., Sykes D. E. (1994). XPS of Oxidized Wool Fibre Surfaces. Surf. Interface Anal..

[cit49] Abo El-Ola S. M., Elsayed N. A. A. (2022). Utilization of Keratin Hydrolysate of Wool Waste Fiber for Free-Salt Dyeing of Viscose Fabric. J. Eng. Fibers Fabr..

[cit50] Ko J., Nguyen L. T. H., Surendran A., Tan B. Y., Ng K. W., Leong W. L. (2017). Human Hair Keratin for Biocompatible Flexible and Transient Electronic Devices. ACS Appl. Mater. Interfaces.

[cit51] Bhardwaj N., Sow W. T., Devi D., Ng K. W., Mandal B. B., Cho N.-J. (2015). Silk Fibroin–Keratin Based 3D Scaffolds as a Dermal Substitute for Skin Tissue Engineering. Integr. Biol..

[cit52] Wijesena R. N., Tissera N. D., Rathnayaka V. W. S. G., Rajapakse H. D., de Silva R. M., de Silva K. M. N. (2020). Shape-Stabilization of Polyethylene Glycol Phase Change Materials with Chitin Nanofibers for Applications in “Smart” Windows. Carbohydr. Polym..

