# Supplementary material for: Keratin protein nanofibers from merino wool yarn: a top-down approach for the disintegration of hierarchical wool architecture to extract α-keratin protein nanofibers
Source: RSC Adv. 2024 Feb 23;14(10):6793–804. doi: 10.1039/d3ra07063h (PMC10885782; doi:10.1039/d3ra07063h)
Supplement: RA-014-D3RA07063H-s001 [file RA-014-D3RA07063H-s001.pdf]

Electronic Supplementary Material (ESI) for RSC Advances.
This journal is © The Royal Society of Chemistry 2024

Supporting information

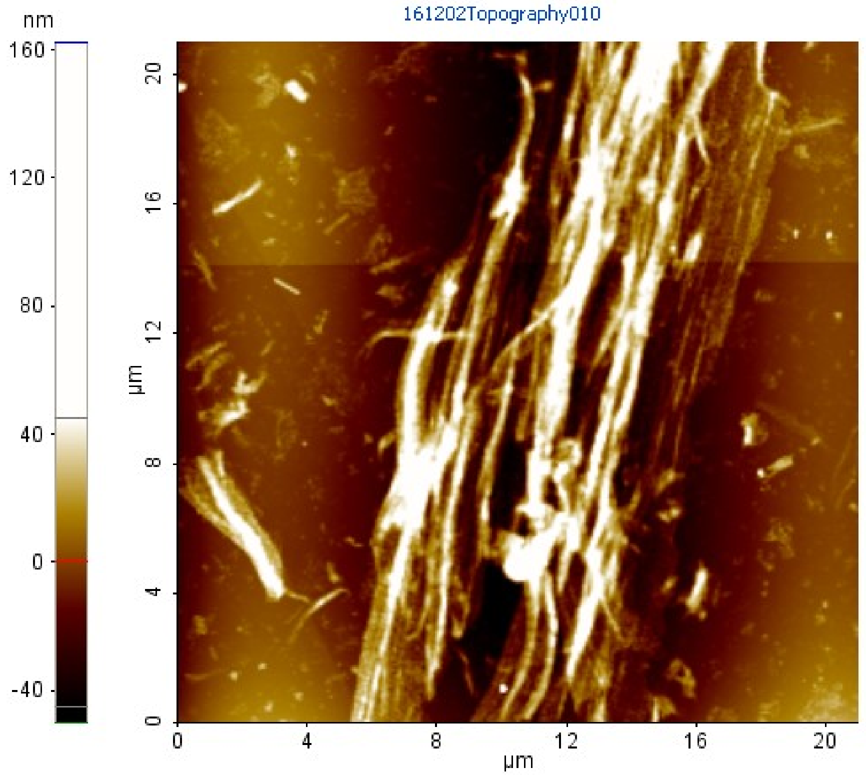

Figure S1: AFM topographical image of the 5 M wool samples subjected to direct sonication in 1.5% acetic acid medium

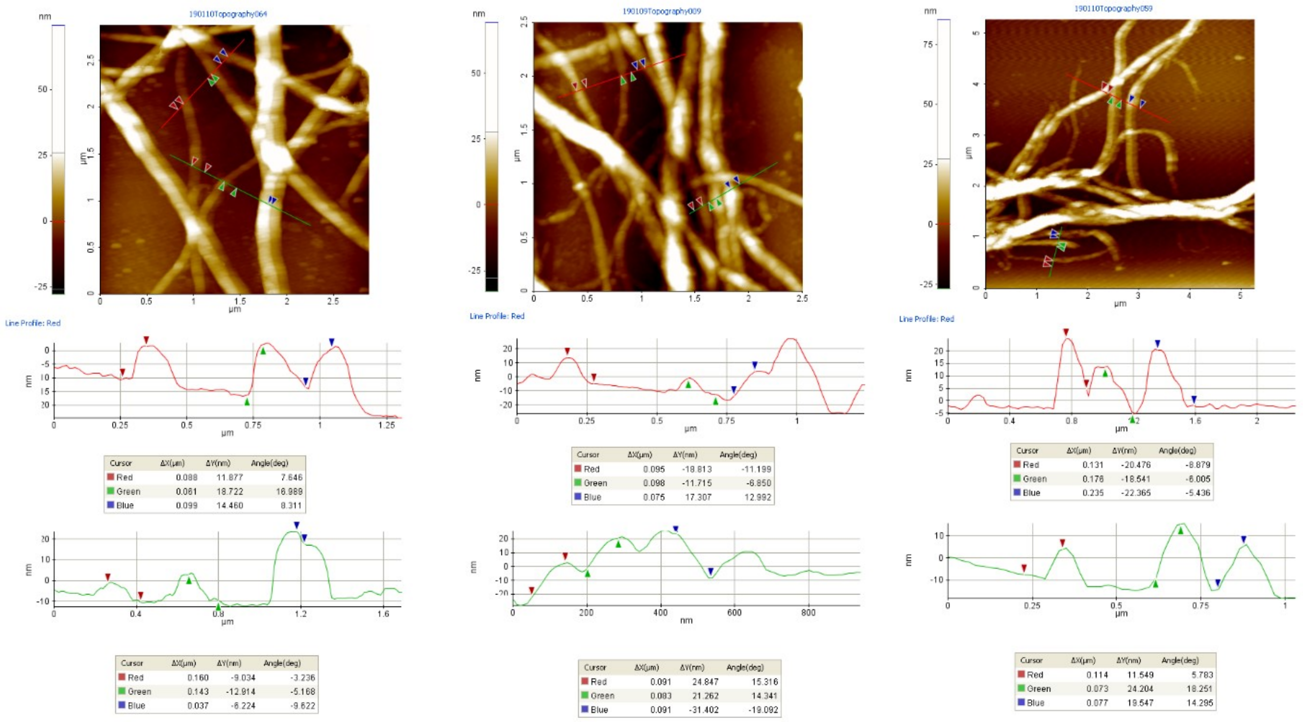

Figure S2: AFM topographical image of the protein nano fibers obtained by subjecting the 5 M wool to high speed blending in 1.5% acetic acid medium

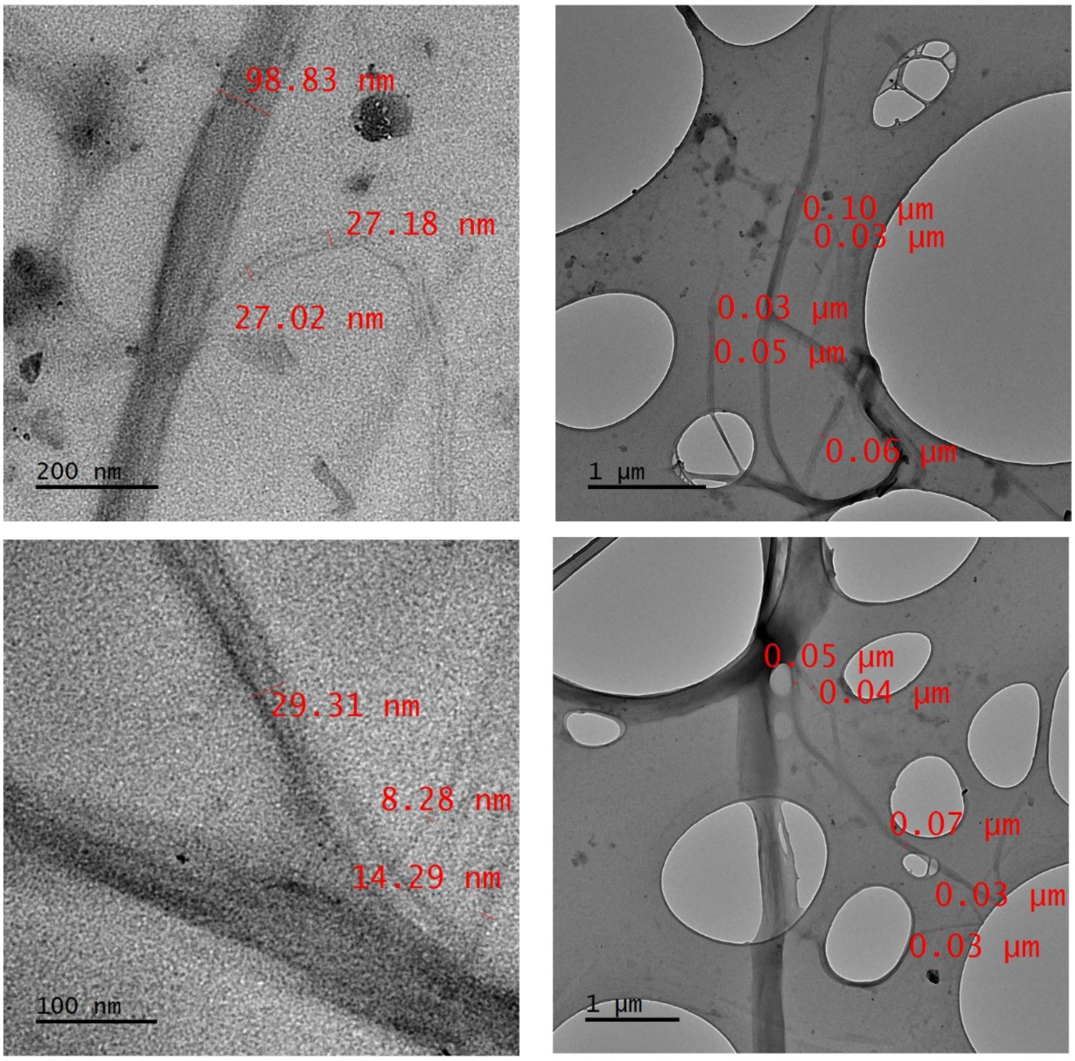

Figure S3: TEM images of the protein nano fibers obtained by subjecting the 5 M wool to high speed blending in 1.5% acetic acid medium

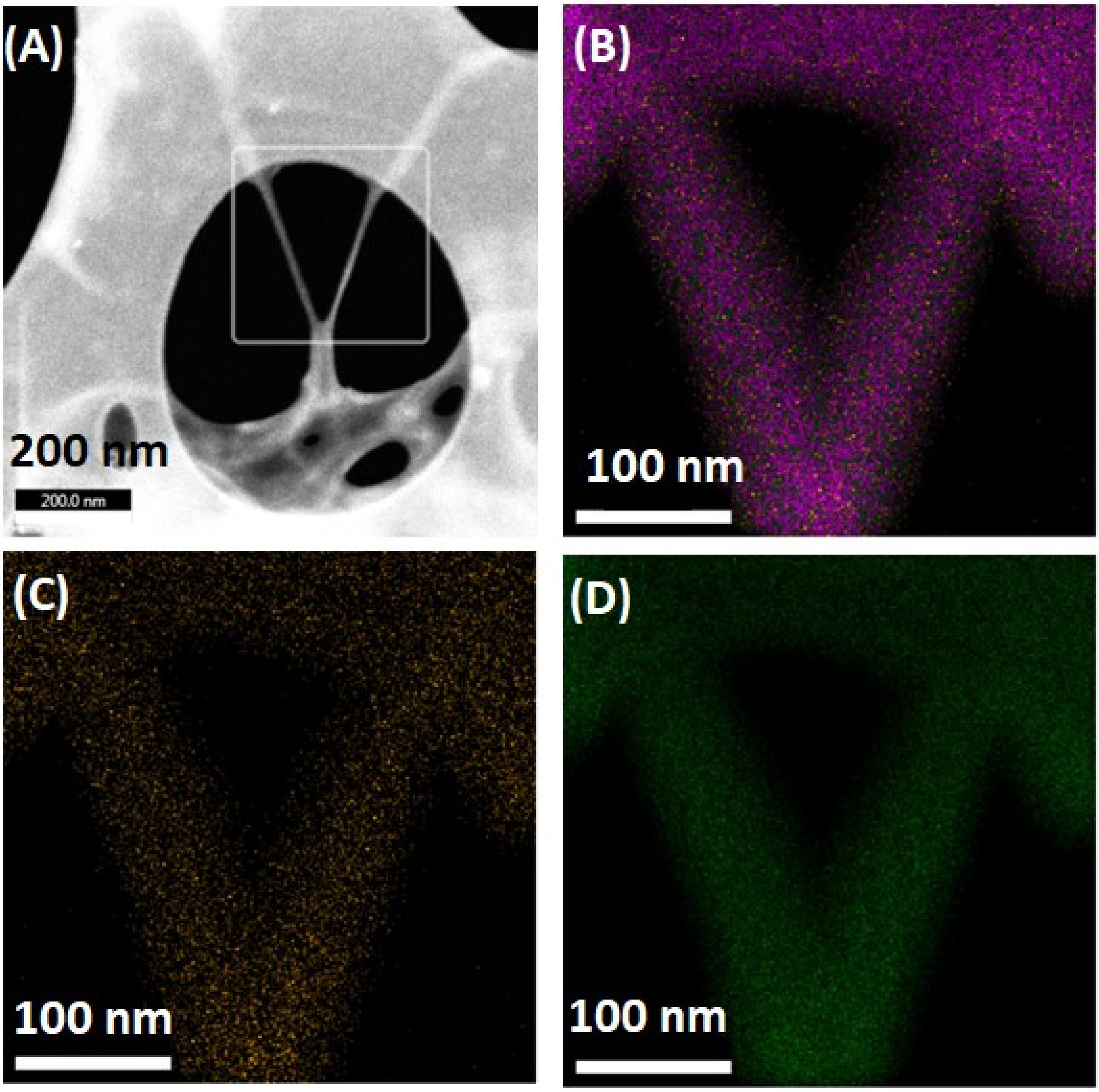

Figure S4: TEM EDX analysis of the protein nano fibers (A) Gray map (B) C map (C) N map (D) O map
